# Impairments in fine motor skills in children with Acute Lymphoblastic Leukaemia. A cross-sectional study

**DOI:** 10.1186/s12887-023-04316-3

**Published:** 2023-10-16

**Authors:** Xochiquetzalli Tejeda-Castellanos, Carlos Maximiliano Sánchez-Medina, Horacio Márquez-González, José Luis Alaniz-Arcos, Ma. Elena Ortiz-Cornejo, Juliette Marie Brito-Suárez, Luis Juárez-Villegas, Claudia Gutiérrez-Camacho

**Affiliations:** 1 Physiotherapy Research Unit, Faculty of Medicine, Universidad Nacional Autónoma de México, Dr. Márquez 162 Colonia Doctores , Mexico City, 06720 Mexico; 2 Research Department, Hospital Infantil de México Federico Gómez, Mexico City, Mexico; 3 Haematology and oncology department of the Hospital Infantil de Mexico, Federico Gómez, Mexico City, Mexico

**Keywords:** Acute Lymphoblastic Leukaemia, Child development disorder, Coordination impairment, Fine motor skills, Leukaemia, Vincristine

## Abstract

**Aim:**

We evaluated fine motor skills; precision, motor integration, manual dexterity, and upper-limb coordination according to sex and risk stratification in children with Acute Lymphoblastic Leukaemia (ALL).

**Methods:**

We evaluated twenty-nine children in the maintenance phase aged 6 to 12 years with the Bruininks-Oseretsky Test of Motor Proficiency-second edition (BOT-2), and sex and age-specific norm values of BOT-2 were used to compare our results.

**Results:**

We found lower scores on the upper-limb coordination subtest, p = 0.003 and on the manual coordination composite, p = 0.008, than normative values. Most boys performed “average” on both the subtests and the composites, but girls showed lower scores with a mean difference of 7.69 (95%CI; 2.24 to 3.14), p = 0.009. Girls’ scale scores on the upper-limb coordination subtest were lower than normative values, with mean difference 5.08 (95%CI; 2.35 to 7.81), p = 0.006. The mean standard score difference in high-risk patients was lower than normative on the manual coordination composite, 8.18 (95%CI; 2.26 to 14.1), p = 0.015. High-risk children also performed below the BOT-2 normative on manual dexterity 2.82 (95%CI; 0.14 to 5.78), p = 0.035 and upper limb coordination subtest 4.10 (95%CI; 1.13 to 7.05), p = 0.028. We found a decrease in fine motor precision in children with a higher BMI, rho= -0.87, p = 0.056 and a negative correlation between older age and lower manual dexterity, r= -0.41 p = 0.026; however, we did not find any correlation with the weeks in the maintenance phase.

**Conclusions:**

Fine motor impairments are common in children with ALL in the maintenance phase; it is important to identify these impairments to early rehabilitation.

**Supplementary Information:**

The online version contains supplementary material available at 10.1186/s12887-023-04316-3.

## Introduction

Acute Lymphoblastic Leukaemia (ALL) is the most common cancer in childhood [1]. Advances in chemotherapy treatment in these patients have increased their survival [2, 3]. However, neurotoxicity from the chemotherapy received has also increased the risk of developing peripheral neuropathy in these children, affecting their fine motor skills [4, 5].

Some fine motor skills affected by peripheral neuropathy are weakness of the distal muscles and decreased strength in the upper and lower limbs, alterations in hand coordination, and sensory disturbances [5–7].

The above fine motor skills in children are essential for developing physical, social and academic activities [8, 9]. Manual skills are developed in the school stage, as well as some others [10–12]. Patients with ALL are often forced to drop out of school for different reasons, including academic ones related to the impairment of these manual skills [13, 14].

Some researchers [15–17] have reported fine motor skills impairments in speed and automation of movement and manual dexterity in children and adolescents with ALL in surveillance and in the treatment phase. De Luca et al. (2013) [18] also studied gross and fine motor skills in children with ALL under surveillance; however, they found no differences in their fine motor skills scores when compared to normative values.

Besides, gender differences in fine motor skills have also been described in children with ALL with low-risk and standard-risk ALL in the maintenance phase, and their results showed girls had the most significant deficit than boys except in fine motor integration and fine motor control composite below the average normative data [19].

Some authors have reported girls’ advantages in graphomotor tasks [20] and learning novel tasks [21]. At the same time, other authors have reported that healthy boys outperform better than girls in tasks that require control of objects, such as throwing objects, while prepubertal girls have better manual control when performing novel tasks [22]. Children with ALL, especially those at high risk, do not manage to have manual control even when they reach preadolescence [23].

With everything described above, the importance of evaluating fine motor skills in children with ALL is clear. Therefore, we aimed to describe alterations in fine motor skills (precision, integration, manual dexterity, and coordination of upper limbs) in schoolchildren with ALL during their treatment and if characteristics such as sex, type of risk, age, weight and weeks of treatment were associated with these impairments.

## Materials and methods

This cross-sectional study was performed between July 2019 to August 2022 in the Hospital Infantil de México, Federico Gómez in Mexico City. This study was part of the protocol approved by the Ethics and Research Committee with register number HIM-2019/078. We invited and recruited children with ALL in the Haematology and Oncology Department who received chemotherapy treatment according to the St. Jude total therapy protocol [24]. We obtained informed consent from all parents/legal guardians and children less than 18 years.

### Inclusion criteria

Boys and girls with ALL aged 6 to 12 years in the maintenance phase at the time of assessment and high and standard risk were included.

### Exclusion criteria

We excluded children with a genetic disorder, pre-existing neurological conditions, those who received cranial radiotherapy, or those who were enrolled in previous or current physical or occupational therapy.

### Sample size calculation

We used a mean difference formula with a power of 80% to determine the sample size. Considering a two-tailed hypothesis, the sample calculated was 26 participants. The type of sampling was for convenience of consecutive cases.

### Participants selection process

**Fig. 1:**
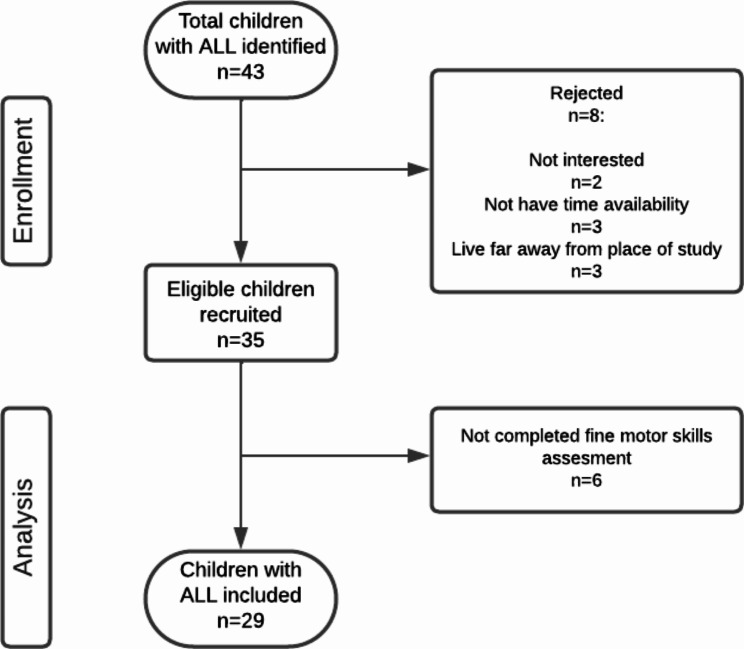
Flow diagram of the progress through the stages of the study

Forty-three children were invited, but eight rejected our invitation because they were uninterested. Thirty-five children with ALL met eligibility criteria, but only 29 were included in the analysis because they completed their fine motor skills assessment (Fig. 1).

### Assessment instrument

We used the BOT-2 ® to assess fine motor skills. The BOT-2 fine motor composite consists of four subtests: fine motor precision, fine motor integration, manual dexterity, and upper-limb coordination, grouped into two composites: fine manual control composite and manual coordination composite. Fine manual control encompasses control and coordination of the distal musculature of the hands and fingers during activities like grasping, writing and drawing. In contrast, manual coordination encompasses control and coordination of the arms and hand in object manipulation [25].

The BOT-2 have an internal consistency between 0.73 and 0.89 in both composites and subtests, with interrater reliability coefficients of 0.98 and 0.99 for the subtests [25].

### Measures and procedures

Once the children were recruited, a physiotherapist with five years of professional practice and training in managing the BOT-2 applied and analysed the tests and subtests to assess fine motor skills. Each child was evaluated individually and required about 20–30 min to complete the subtests.

Participants were required to use their dominant drawing hand for all subtests except the upper limb coordination subtest, performed by the dominant throwing hand according to the BOT-2 manual.

Subsequently, children’s clinical characteristics such as sex, age, weeks on the maintenance phase at assessment, weight, height and risk stratification (standard, high), weight z-score classification and comorbidities were registered. Anthropometric measures were determined by a trained nutritionist using a Medical beam scale for weight, and Stadiometer Nuevo León S.A de C.V for height. Weight z-score classification was determined according to the World Health Organization (WHO).

Finally, we represented scale and standard scores for composites and subtests in descriptive categories ranging from “Well-below average” to “Well-above average.“ The BOT-2 manual defines the “Average” category as ± 1 standard deviation of the scale or standard score [25]. Additionally, reported total point scores according to age- and sex-specific scale scores (X = 15; SD = 5) and standard scores (X = 50; SD = 10) according to the BOT-2 normative reference [25].

### Statistical analysis

The Shapiro-Wilks test was used to determine the data normality. Mean and standard deviation (± SD) were used to represent our quantitative variables (age, weight, height and weeks on maintenance phase) and frequencies relatives and absolutes were used to describe the qualitative variables (stratification risk, comorbidities, z score classification).

We used a one-sample independent *t-test* to compare the reference value in BOT-2 subtests and composite scores and the U-Mann Whitney test to compare differences between sex and risk stratification. The Pearson correlation coefficient was calculated to determine the association between BOT-2 subtests, composite scores, weeks on maintenance phase and age. The Spearman correlation coefficient was also calculated to determine the association between BOT-2 subtests and composite scores and qualitative variables (sex, risk stratification and weight z-score classification).

We compare the mean score standard differences between the scholarship included and the BOT-2 normative with 95% confidence intervals and significance level < 0.05. The statistical analysis was performed using the Stata program version 15.1 for Windows (StataCorp, Texas, USA), and the graphs were made using GraphPad Prism 8 (GraphPad Software, San Diego, CA, USA).

## Results

We included 29 children aged 7.9 ± 1.29 years, and 16 were boys (55.2%). Clinical characteristics showed that boys had greater body weight (p > 0.05) and girls were stratified with a higher risk of disease (p = 0.96). In addition, only one girl had a history of COVID-19, and another had lower limb neuropathy. Therefore, four children had a relapse history, and three had high-risk ALL (two boys and one girl) (Table 1).

**Table 1:** Clinical characteristics of the children

Characteristics n (%)	Boys 16 (55.17)	Girls 13 (44.83)	p-value
Age (years), mean ± SD	7.83 ± 1.20	7.91 ± 1.45	0.87^†^
Weeks on maintenance phase, mean ± SD	46.44 ± 30.46	56.46 ± 40.91	0.46^†^
Weight (kg), mean ± SD	24.18 ± 5.11	23.55 ± 5.55	0.75^†^
Height (cm), mean ± SD	121.07 ± 9.80	121.31 ± 12.63	0.95^†^
Weight Z score classification, n (%)			
Obesity Overweight Normal weight Underweight	2 (13.3) 2 (13.3) 10 (66.6) 1 (6.6)	0 1 (7.6) 11 (84.6) 1 (7.6)	0.49^∞^ 1.0^∞^ 0.23^∞^ 1.0^∞^
Risk stratification, n (%)			
High Standard	6 (37.5) 10 (62.5)	8 (61.5) 5 (38.5)	0.96^††^
Hand dominant for drawing and throwing, n (%)			
Right	15 (93.75)	12 (92.3)	1.0^∞^

^†^t-student; ^∞^Fisher’s exact test; ^††^chi-squared test

### Fine manual control and manual coordination composites

**Fig. 2:**
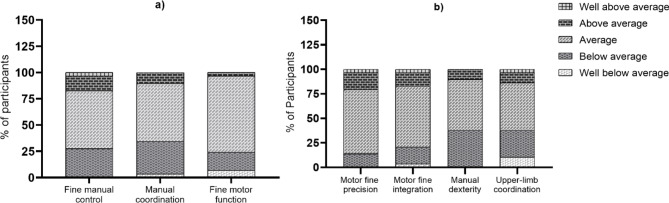
(a) Descriptive categories on BOT-2 composites of children with ALL. (b) Descriptive categories on BOT-2 subtests of children with ALL

The descriptive categories for fine manual control and manual coordination composites are shown in Fig. 2a. Most of the children had an “average” performance; however, almost 30% of them were categorised as “below average” or “well-below average” performance, and the manual coordination composite had the highest proportion of children with “below average”.

**Fig. 3:**
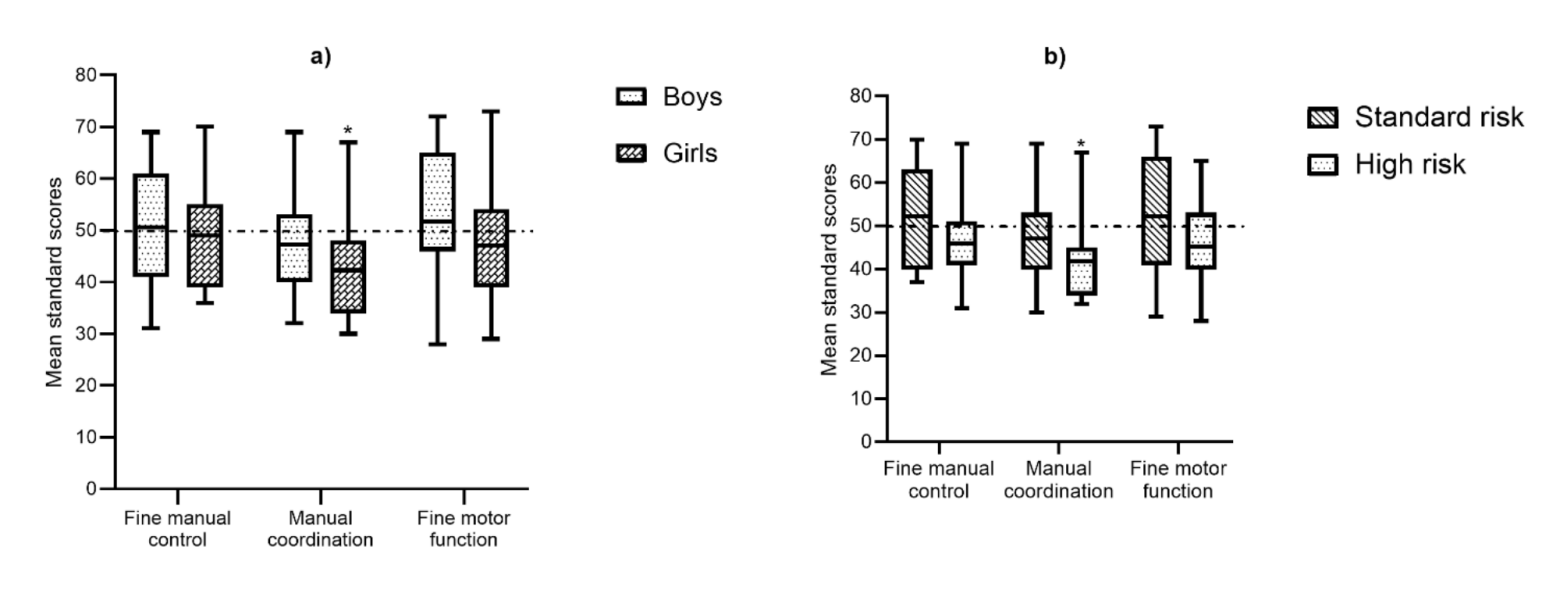
(a) Mean differences in BOT-2 composites according to sex. (b) Mean differences in BOT-2 composites according to risk stratification. *Statistically significant (p < 0.05). BOT-2 Standard score mean = 50 (SD = 10) [25]

The mean standard score difference on BOT-2 composites according to sex showed that girls had lower scores than the age-match norm on manual coordination with a mean difference of 7.69 (95% CI 2.24 to 13.14), p = 0.009. The mean standard score difference in high-risk patients was significantly lower than standard-risk patients on the age-matched norm on the manual coordination composite, with a mean difference of 8.18 (95% CI 2.26 to 14.1), p = 0.015. Fine motor control and fine motor function were not statistically significant, with p > 0.05 (Fig. 3a and b).

Although differences previously mentioned compared to the age-match norm, we did not find statistically significant differences between boys and girls in our sample, nor between high-risk and standard-risk stratification (p > 0.05).

We found no correlation between BOT-2 subtests scale scores nor composites standard scores and the weeks on the maintenance phase, risk stratification, nor Z score > 1 (p > 0.05).

### BOT-2 subtests

The descriptive categories for fine motor precision, integration and manual dexterity, and upper-limb coordination subtests are shown in Fig. 2b. Most of the children had an “average” performance; however, almost 30% of them were categorised as having “below average” or “well-below average” performance on manual dexterity and upper-limb coordination. The manual dexterity subtest had the highest proportion of children “below average”.

**Fig. 4:**
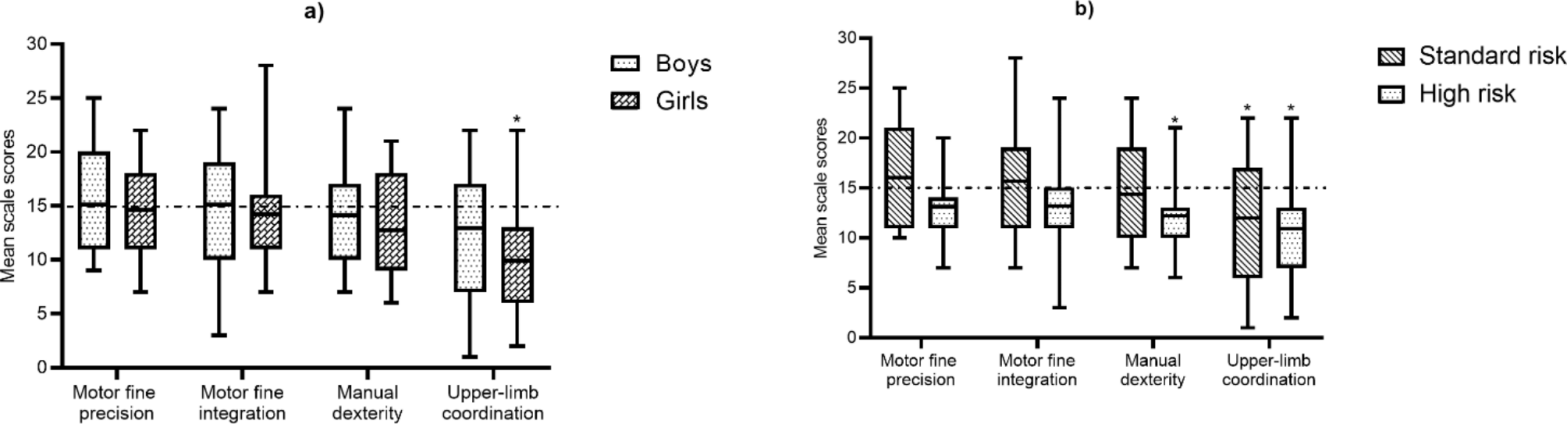
(a) Mean differences on BOT-2 subtests according to sex. (b) Mean differences on BOT-2 subtests according to risk stratification. *Statistically significant (< 0.05). BOT-2 Scale Score mean = 15 (SD = 5) [25]

ALL children included performed more poorly compared to the standardised age-match BOT-2 norm on manual dexterity with a mean difference of 1.48 (95% CI -3.30 to 0.34), p = 0.057, but this value was not statistically significant. However, on upper-limb coordination subtests of the BOT-2 scale scores were significantly lower in ALL studied children with a mean difference of 3.42 (95% CI 1.59 to 5.23), p = 0.0031. Furthermore, girls’ scale scores on the upper-limb coordination subtest were significantly lower than the age-match BOT-2 norm with a mean difference of 5.08 (95% CI 2.35 to 7.81), p = 0.006 (Fig. 4a).

Regarding risk stratification, high-risk children performed below the BOT-2 norm for age in manual dexterity with a mean difference of 2.82 (95% CI 0.14 to 5.78), p = 0.035 (Fig. 4b). In addition, standard-risk and high-risk children had lower scores on the upper limb coordination subtest than the BOT-2 norm; mean differences were 3.0 (95%CI 0.69 to 5.31), p = 0.030 and 4.10 (95%CI 1.13 to 7.05), p = 0.028 respectively (Fig. 4b).

Fine motor precision and fine motor integration subtests were not different from the norm data (p > 0.05). We did not find differences between boys and girls, nor between high-risk and standard-risk patients.

We found a negative correlation between age and manual dexterity scale score (r= -0.41), p = 0.026 (Online Resource 1). We did not find a significant correlation between weight z-score classification and the subtests scale score; however, we found a decrease in fine motor accuracy in children with the highest BMI (rho= -0.87), p = 0.058.

## Discussion

The main findings of our study were that manual coordination composite in children with ALL evaluated was the most affected fine motor skills as well as upper limb coordination and manual dexterity domains in girls and high-risk children.

Fine motor skills are particularly important when children are at the school stage, where they spend more than half of the day completing academic tasks that require the integrity of these skills [26]. In particular, the completeness of hand coordination and its subtests allow for goal-directed activities involving reaching, grasping, and hand coordination to pick up small objects and for self-care activities such as holding eating utensils. In older children, when these skills are altered, the quality of writing may be affected, leading to poor student performance [27].

Additionally, alterations in fine motor skills could affect the social, academic, communication, and coping environment of children with cancer [13, 28–30].

Our study showed lower scores in girls in upper limb coordination, which are relevant skills for catching and throwing objects; however, this finding differs from that reported by Hamari L et al. (2020), [29] who described better scores in girls in manual dexterity and coordination of upper limbs when they were evaluated at 6, 12, and 30 months (p > 0.05). An explanation for these differences is that, in our study, most of the affected girls were classified as high-risk. In contrast, the affected girls reported in Hamari’s study had low-risk or standard risk. We consider this finding relevant because children with high-risk ALL who receive frequent, high doses of chemotherapeutic agents have been reported to be at increased risk of developing fine motor deficits; however, our study could not demonstrate this [23, 30].

In addition, the children evaluated in our study were older than those included by Hamari et al. [29], who included children aged 5.62 ± 1.11 years. This aspect is relevant since school-age children, like those in our research, perform tasks that require more complex manual and upper limb coordination than younger children, which, in addition to making these motor deficiencies more evident, could explain the negative correlation found in our study between older age and lower manual dexterity.

On the other hand, sex has been described as another factor in explaining motor skills in boys, which can somehow explain the poor performance of our girls due to gender stereotypes about the type of games and activities in which they should participate [20].

Besides, our study showed that children with higher BMI had poor fine motor precision (rho= -0.87), p = 0.058. This finding has already been reported; an example of this is the study of Gentier et al. (2013), [31] who reported worse scores in fine motor precision and manual dexterity according to their weight increase in patients aged 7–13 years. Our results suggest a trend towards poorer motor performance in overweight patients compared to normal-weight patients; however, our sample size was small.

Our study has strengths, such as identifying fine motor skills impairment in older girls with ALL patients with high risk during the maintenance phase. However, despite the strengths of our study, we also recognise several limitations related to the small sample size, the cross-sectional design, and its consequent selection bias; therefore, we did not search for a correlation between chemotherapy and fine motor impairments.

This study highlighted the importance of evaluating fine motor skills in children with cancer, specifically those girls with Acute Lymphoblastic Leukemia. The early identification of deterioration in manual coordination and its upper limbs is essential for the treating physicians to refer children promptly for rehabilitation. This timely rehabilitation will allow these children to achieve their social, academic and recreational activities necessary for optimal growth and development.

## Conclusion

In the maintenance phase, fine motor impairments are common in children with ALL, and their identification could facilitate early rehabilitation.

### Electronic supplementary material

Below is the link to the electronic supplementary material.

Supplementary Material 1

## Data Availability

All data generated or analysed during this study are included in this published article and its supplementary information files.

## Abbreviations

ALL: Acute Lymphoblastic Leukaemia
BOT-2: Bruininks-Oseretsky Test of Motor Proficiency- second edition
WHO: World Health Organization
