# Supplementary material for: Impairments in fine motor skills in children with Acute Lymphoblastic Leukaemia. A cross-sectional study
Source: BMC Pediatr. 2023 Oct 16;23:513. doi: 10.1186/s12887-023-04316-3 (PMC10578039; doi:10.1186/s12887-023-04316-3)
Supplement: Supplementary file 1 — Supplementary Material 1 [file 12887_2023_4316_MOESM1_ESM.docx]

**Impairments in fine motor performance in children with acute lymphoblastic leukemia. A cross-sectional study**

Xochiquetzalli Tejeda-Castellanos^a^ PT, Carlos Maximiliano Sánchez-Medina^a^ PT, Horacio Márquez-González^b^ PhD, José Luis Alaniz-Arcos^a^ MD, Ma. Elena Ortiz-Cornejo^a^ MD, Juliette Marie Brito-Suárez^a^ MD, Luis Juárez-Villegas^c^ MD, Claudia Gutiérrez-Camacho^a^ PhD

^a^Physiotherapy Research Unit, Faculty of Medicine, Universidad Nacional Autónoma de Mexico, Mexico City, México

^b^Research Department, Hospital Infantil de México Federico Gómez, Mexico City, Mexico

^c^ Haematology and oncology department of the Hospital Infantil de Mexico, Federico Gómez

**Corresponding author:**

Claudia Gutiérrez-Camacho, MD, PhD, Physiotherapy Research Unit. Faculty of Medicine, Universidad Nacional Autónoma de México. Dr. Márquez 162 Colonia Doctores CP 06720 Mexico City. Address all correspondence to Dr. Gutiérrez at: claudia.g.cam@facmed.unam.mx


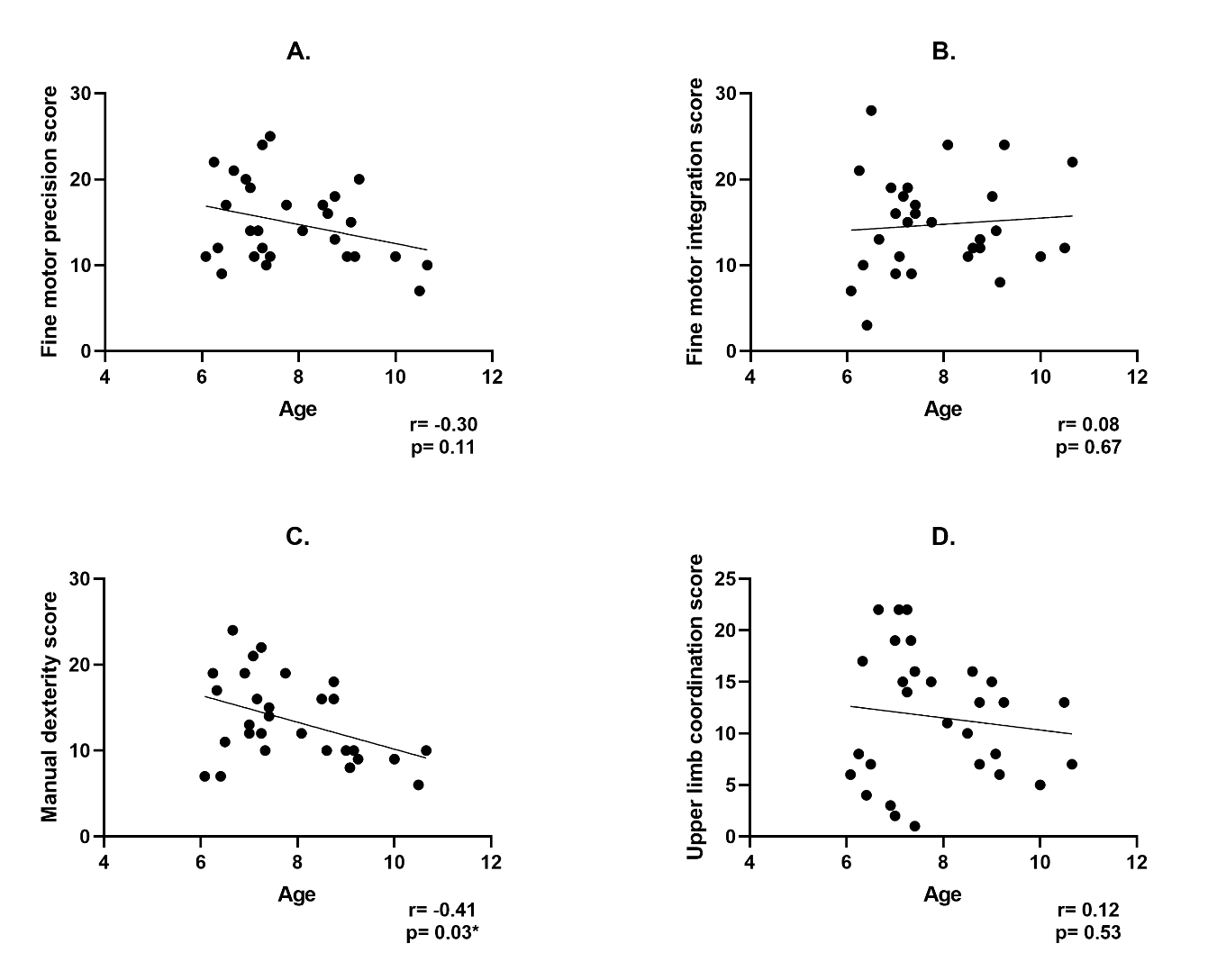


**Online resource 1 A)** Pearson correlation between fine motor precision and age. **B)** Pearson correlation between fine motor integration and age. **C)** Pearson correlation between manual dexterity and age. **D)** Pearson correlation between upper-limb coordination and age. *Statistically significant (<0.05)
